# Weed Mapping in Early-Season Maize Fields Using Object-Based Analysis of Unmanned Aerial Vehicle (UAV) Images

**DOI:** 10.1371/journal.pone.0077151

**Published:** 2013-10-11

**Authors:** José Manuel Peña, Jorge Torres-Sánchez, Ana Isabel de Castro, Maggi Kelly, Francisca López-Granados

**Affiliations:** 1 Department of Crop Protection, Institute for Sustainable Agriculture (IAS) Spanish National Research Council (CSIC), Córdoba, Spain; 2 Environmental Science, Policy and Management Department, University of California, Berkeley, California, United States of America; Universidad de Castilla-La Mancha, Spain

## Abstract

The use of remote imagery captured by unmanned aerial vehicles (UAV) has tremendous potential for designing detailed site-specific weed control treatments in early post-emergence, which have not possible previously with conventional airborne or satellite images. A robust and entirely automatic object-based image analysis (OBIA) procedure was developed on a series of UAV images using a six-band multispectral camera (visible and near-infrared range) with the ultimate objective of generating a weed map in an experimental maize field in Spain. The OBIA procedure combines several contextual, hierarchical and object-based features and consists of three consecutive phases: 1) classification of crop rows by application of a dynamic and auto-adaptive classification approach, 2) discrimination of crops and weeds on the basis of their relative positions with reference to the crop rows, and 3) generation of a weed infestation map in a grid structure. The estimation of weed coverage from the image analysis yielded satisfactory results. The relationship of estimated versus observed weed densities had a coefficient of determination of r^2^=0.89 and a root mean square error of 0.02. A map of three categories of weed coverage was produced with 86% of overall accuracy. In the experimental field, the area free of weeds was 23%, and the area with low weed coverage (<5% weeds) was 47%, which indicated a high potential for reducing herbicide application or other weed operations. The OBIA procedure computes multiple data and statistics derived from the classification outputs, which permits calculation of herbicide requirements and estimation of the overall cost of weed management operations in advance.

## Introduction

Many agricultural crops require the use of herbicides as essential tools for maintaining the quality and quantity of crop production. Currently, the cost of herbicides accounts for approximately 40% of the cost of all the chemicals applied to agricultural land in Europe [1]. Associated environmental and economic concerns have led to the creation of European legislation on the sustainable use of pesticides [2]. This legislation includes guidelines for the reduction in applications and the utilization of adequate doses based on the degree of weed infestation. Both components are integrated in the agronomical basis of the precision agriculture principles and especially of site-specific weed management (SSWM). This consists ofthe application of customized control treatments, mainly herbicides, only where weeds are located within the crop field in order to use herbicides and doses according to weed coverage [3]. SSWM typically uses new technologies to collect and process spatial information on the crop field. Remote sensing technology can play a role here as an efficient and repeatable method to obtain crop field information related to weed infestation. 

The analysis of remote images captured with aircraft and satellite platforms has resulted in numerous examples of weed mapping in late growth stages [4-6], although in many weed–crop systems, the optimal treatment time is early in the growth season when weeds and crops are in their seedling growth stages [7]. However, discriminating small seedlings with airborne and satellite imagery is problematic due to the insufficient spatial resolution of these images. This difficulty might be now overcome using the new generation of remote platforms known as unmanned aerial vehicles (UAV) or unmanned aerial systems (UAS). UAVs can operate at low altitudes and capture images at very high spatial resolutions (a few cm), which is not feasible with conventional remote platforms. Moreover, UAVs can work on demand with great flexibility at critical moments, depending on the agronomic goals involved. This is crucial for detecting small weed and crop plants at early stages in the majority of fields. UAV technology has been adapted and utilized by diverse groups interested in agricultural investigation [8], and a few studies have reported the use of UAVs in assessing weed distribution or invasion of plants in rangeland monitoring [9,10].

Along with spatial and temporal resolution requirements, spectral similarity between weed and crop plants, which occurs mainly in the early part of the growth season, makes discrimination between the two difficult [7,11]. This is an important limitation in the application of image analysis methods based on pixel information only. To address this limitation, a powerful procedure, such as object-based image analysis (OBIA) might be the only way to distinguish between weed and crop. The OBIA methodology first identifies spatially and spectrally homogenous units (objects) created by grouping adjacent pixels according to a procedure known as segmentation and next it combines spectral, contextual and morphological information to drastically improve image classification results [12]. In this process, the definition of the row structure formed by the crop is essential for further identification of plants (crop and weeds) because the position of each plant relative to the rows might be the key feature used to distinguish among the weeds and crop plants [13]. 

In the context of SSWM, the ultimate objective of detecting weed patches is to generate efficient decision support system data that can be used with specific spraying machinery [14]. For this purpose, several applications have been developed to delineate a restricted number of management zones based on crop status [15] or weed density thresholds in mature wheat fields [16]. However, the development of robust and automatic procedures for weed data acquisition, image analysis and delineation of weed cover zones is still challenging, even more so in early growth stages [7]. This research involves the whole process: acquisition of very-high-spatial-resolution remote images with a UAV, image analysis using object-based methods, and the ultimate objective of generating weed maps at early stages for in-season site-specific herbicide treatment. To achieve this objective, we developed an OBIA procedure consisting of three main phases: 1) automatic definition of crop rows within a maize field accomplished by combining spectral and contextual features in a customized looping rule set algorithm, 2) discrimination of weed seedlings and crop plants based on their relative positions, and 3) automatic generation of a weed coverage map in a grid framework adapted to the specification required by the herbicide spraying machinery.

## Materials and Methods

### Study site

**Figure 1:**
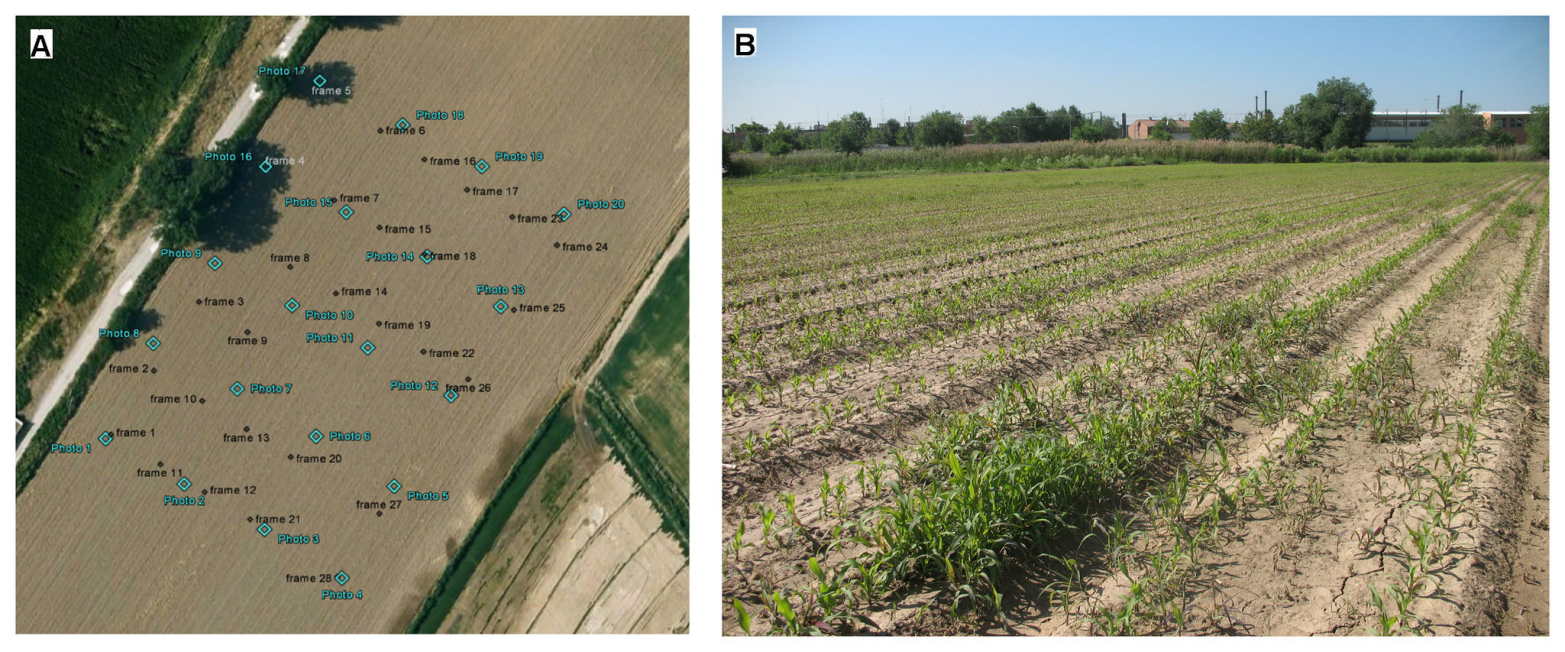
Aerial view of the experimental field (a), showing the centers of the UAV aerial images in blue and the sampling points in black (see section 2.4), and in-field photograph of the study site (b), showing the maize rows and some patches of weed infestation.

Remote images were taken on May 5^th^, 2011 on a maize field located in Arganda del Rey (Madrid, Spain, coordinates 40.320 N, 3.477 W, datum WGS84), just when post-emergence herbicide or other control techniques are recommended. The flights were authorized by a written agreement between the farm owners and our research group. The maize field was naturally infested with *Amaranthus blitoides* (broad-leaved weed) and *Sorghum halepense* (grass weed). The maize was at the stage of 4–6 leaves unfolded, and the weed plants were similar in size or in some cases smaller than the maize plants (Figure 1). Several visits to the field were conducted for monitoring of crop growth and weed emergence and finally to select the best moment to take the set of remote images. An experimental plot of 140x100 m was delimited within the crop field to perform the flights. The coordinates of each corner of the flight area were collected with a global positioning system (GPS) for use in planning the flight route. 

### UAV flights and remote images

**Figure 2:**
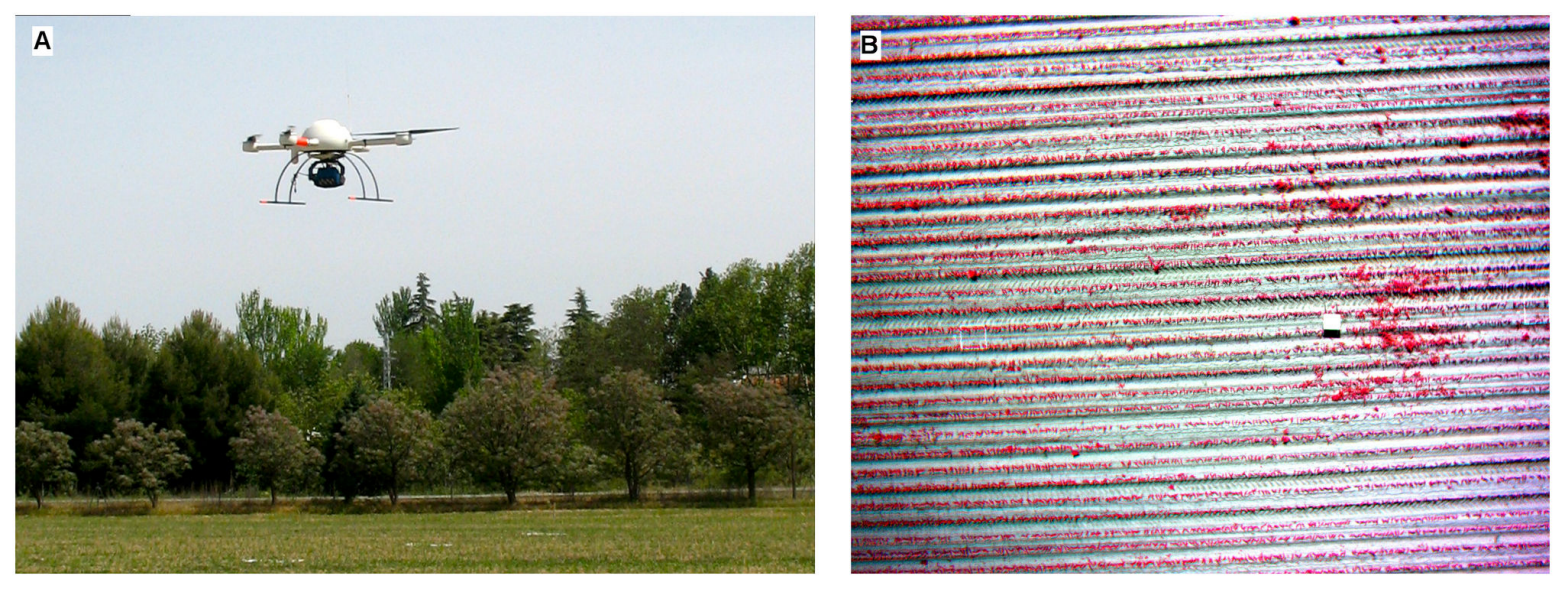
Unmanned quadrotor-type aerial vehicle flying over the crop field (*a*), and aerial image (color–infrared composition) obtained by the UAV at an altitude of 30 m (*b*), showing the maize rows, some weed patches and the Spectralon^®^ panel.

A model md4-1000 quadrocopter UAV (microdrones GmbH, Siegen, Germany) with vertical take-off and landing capabilities was used to collect the remote images (Figure 2a). This UAV can fly either by remote control or autonomously with the aid of its GPS receiver and its waypoint navigation system. It can carry any sensor that weighs less than 1.25 kg mounted under its belly. The images were collected with a Tetracam mini-MCA-6 camera (Tetracam Inc., Chatsworth, CA, USA), which is a lightweight (700 g) multispectral sensor with six individual digital channels arranged in a 2x3 array. Each channel has a focal length of 9.6 mm and a 1.3-megapixel (1,280 x 1,024 pixels) CMOS sensor that stores images on a compact flash card. The camera has user-configurable band-pass filters (Andover Corporation, Salem, NH, USA) of 10-nm full width at half-maximum and center wavelengths of 530, 550, 570 (the green region of the electromagnetic spectrum), 670 (the red region), 700 and 800 nm (the near-infrared region). The software PixelWrench2 was supplied with the camera to provide full camera control and image management [17], including correction of the vignette effect, alignment of RAW image sets and building of multi-band TIFs (Figure 2b), as explained in [18]. 

The flight altitude was 30 m above ground level, yielding 20 images of 2-cm spatial resolution to cover the whole experimental field. During the UAV flights, a barium sulphate standard Spectralon® panel (Labsphere Inc., North Sutton, NH, USA) 1 x 1 m in size was placed in the middle of the field to calibrate the spectral data (Figure 2b). Digital images captured by each camera channel were spectrally corrected by applying an empirical linear relationship [19]. Equation coefficients were derived by fitting the digital numbers of the MCA imagery located in the spectralon panel to the spectralon ground values.

### Weed mapping by object-based image analysis (OBIA)

**Figure 3:**
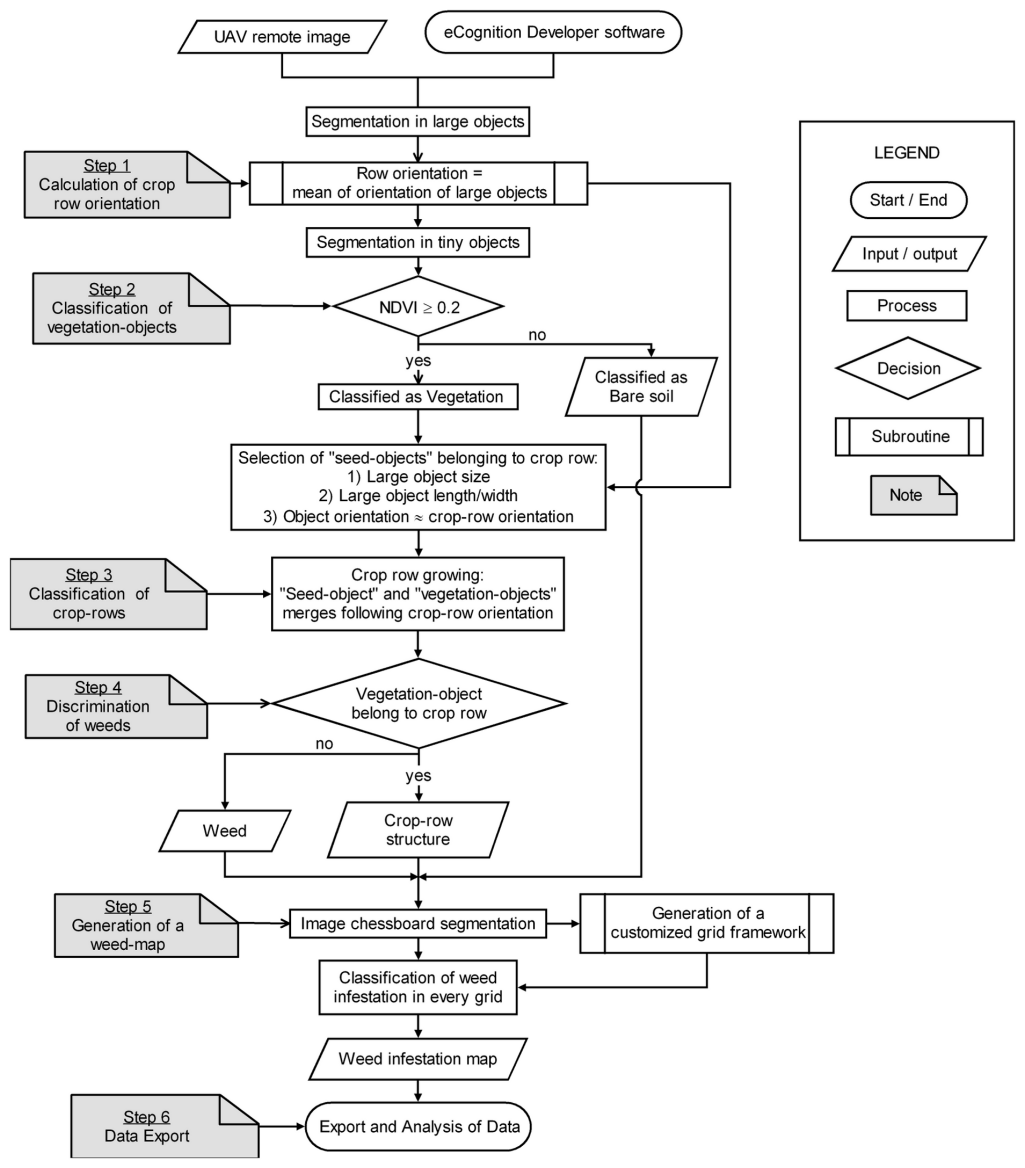
Flowchart of the OBIA procedure for classification of crop rows and weeds and generation of a weed infestation map.

The spectral characteristics and general appearance of crop and weed plants are highly similar in the early season [7,11] and are even more pronounced in remote images [18]. Therefore, the effectiveness of weed discrimination might be increased by taking advantage of the relative position of every plant with reference to the crop row structure [13]. This information can be included in the classification procedure using the OBIA methodology, allowing the combination of spectral, contextual and morphological information, among other features, of the objects created using a procedure known as segmentation [20]. The commercial software eCognition Developer 8 (Trimble GeoSpatial, Munich, Germany) was used to analyze the UAV images and develop an OBIA procedure. The rule set algorithm for weed mapping ran automatically and consisted of three consecutive phases: 1) classification of crop rows, 2) discrimination between crop plants and weeds based on their relative positions, and 3) generation of a weed infestation map in a grid structure. A flowchart of the process is shown in Figure 3.

#### Crop row classification

**Figure 4:**
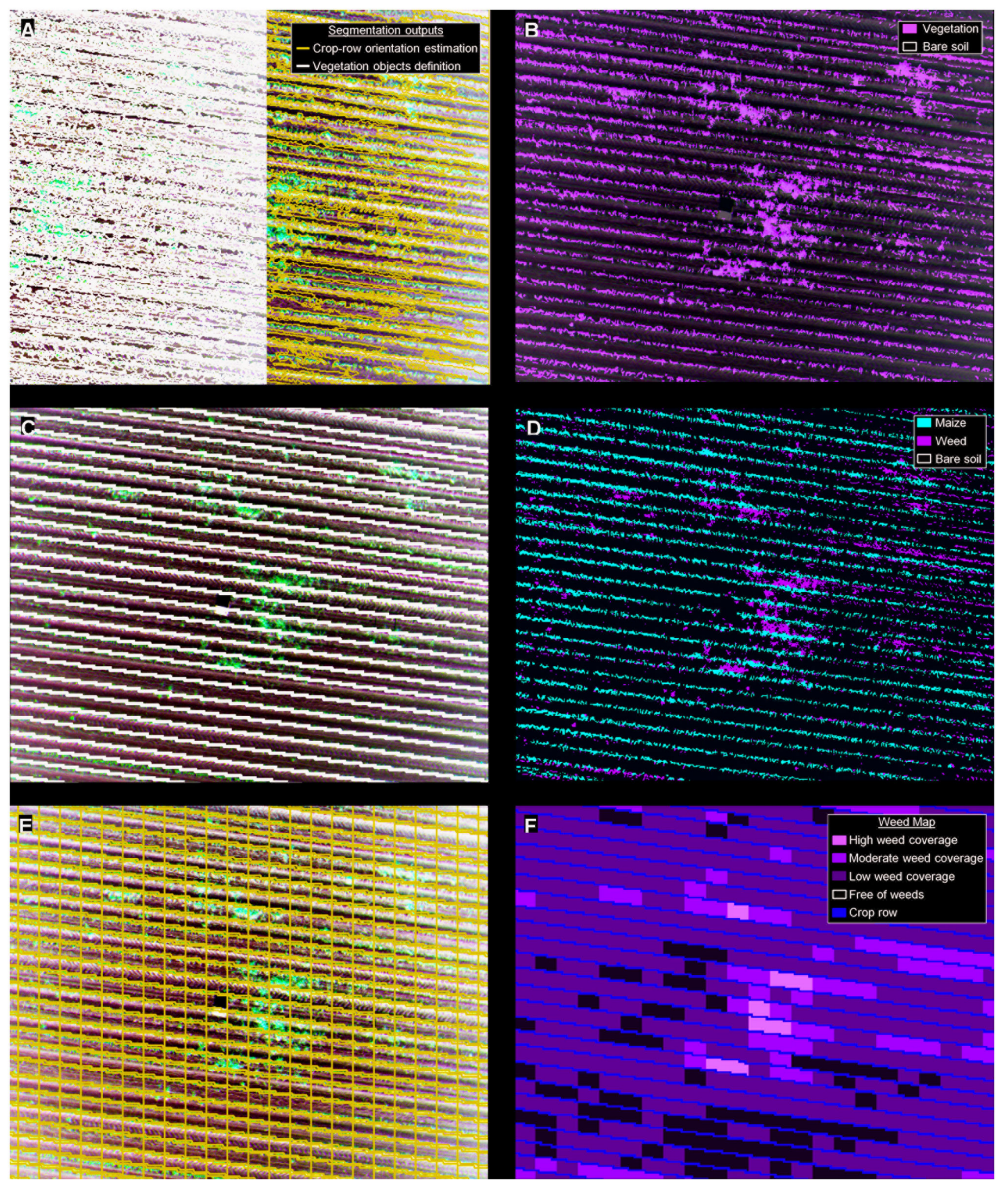
Partial view of the outputs of the OBIA procedure at each step: *a*) segmentation outputs at scales of 140 (in blue) and 10 (in black), used to calculate row orientation and define vegetation objects, respectively; *b*) classification of objects of vegetation and bare soil ; *c*) definition of the crop row structure (in black); *d*) classified image with crop, weeds and bare soil; *e*) grid framework of the inter-row area; *f*) weed coverage map showing three levels of infestation (low, moderate and high), crop rows and weed-free zones.

A dynamic and auto-adaptive classification approach was used to define the crop row structure, by mean of a combination of several object-based features that characterize a set of regular and quasi-equidistant lines of plants. In this process, the UAV images were segmented into homogeneous multi-pixel objects using the multiresolution algorithm [21]. Segmentation is a bottom-up region-merging process in which the image is subdivided into homogeneous objects on the basis of several parameters (band weights, scale, color, shape, smoothness and compactness) defined by the operator [22]. Two levels of segmentation were independently used throughout the procedure (Figure 4a): 1) a level at a scale of 140, to define the main orientation of the crop rows, and 2) a level at a scale of 10, to generate smaller objects for crop and weed discrimination. In both cases, the values of the other parameters involved in the segmentation were 0.9, 0.1, 0.5 and 0.5 for color, shape, smoothness and compactness, respectively. 

After segmentation, the normalized difference vegetation index (NDVI; [23]) was used to classify objects of vegetation (Figure 4b) as being those with NDVI values greater than 0.20. NDVI was selected as the best index for use in performing this classification, compared to other vegetation indices [18]. A customized merging operation was then performed to create lengthwise vegetation objects, following the shape of a crop row. In this operation, two candidate vegetation objects were merged only if the length/width ratio of the target object increased after the merging. Next, the object that was largest in size and with orientation close to the row orientation was classified as a seed object belonging to a crop row. Lastly, the seed object grew in both directions, following the row orientation, and a looping merging process was performed until all the crop rows reached the limits of the parcel (Figure 4c). Every phase of the crop row classification process is described in detail in [24].

#### Discrimination of crop and weeds

After classifying all the crop rows within an image, the algorithm generated a buffer zone along the longitudinal axis of each row by applying a chessboard segmentation process at an upper level of hierarchy. Two or more levels of segmentation form a hierarchical structure in the OBIA paradigm, in which super-objects belong to the upper level and include one or more sub-objects that belong to the lower level. In this case, the width of the buffer zone (upper hierarchical level) was defined by the average size of the vegetation objects in contact with the row structure. Next, the vegetation sub-objects located entirely below the buffer zone (lower hierarchical level) were classified as crop plants, and others were classified as weeds (Figure 4d). A more complex decision rule was made in the case of sub-objects located below the edge of the buffer zone. In this case, the sub-objects in contact with or very close to other weeds were classified as weeds because aggregation among weed plants, i.e., weed patches, was generally observed [25]. 

#### Weed coverage mapping

After weed–crop classification, the algorithm built a grid framework of the inter-row area by applying two consecutive processes: 1) copying the existing inter-row object level to an upper position, and 2) chessboard segmentation of this upper level and generation of grids of user-adjustable size (Figure 4e). For example, in this investigation, the grid length used was 1 m and the grid width used was the inter-row distance (0.7 m on average). Therefore, a new hierarchical structure was generated in the inter-row area between the grid super-objects (upper level) and the weed and bare-soil sub-objects (lower level). Next, an estimate of the weed coverage (% of weeds) was automatically calculated from the ratio of weed pixels to total pixels per grid [13,26]. This calculation was based on the hierarchical relationship between grid super-objects and weed-infested sub-objects. Lastly, weed cover was also mapped on the basis of a number of user-adjustable categories defined by infestation thresholds. For example, in this investigation, the weed map identified both weed-free zones and weed-infested zones, which were categorized at three different levels of infestation, as follows: 1) low (<5% weed coverage), 2) moderate (5–20% weed coverage) and 3) high (>20% weed coverage) (Figure 4f). Both the grid dimensions and the number and thresholds of the weed infestation categories can be customized on the basis of cropping patterns and the specifications required by the herbicide spraying machinery. 

### The evaluation of the methodology

**Figure 5:**
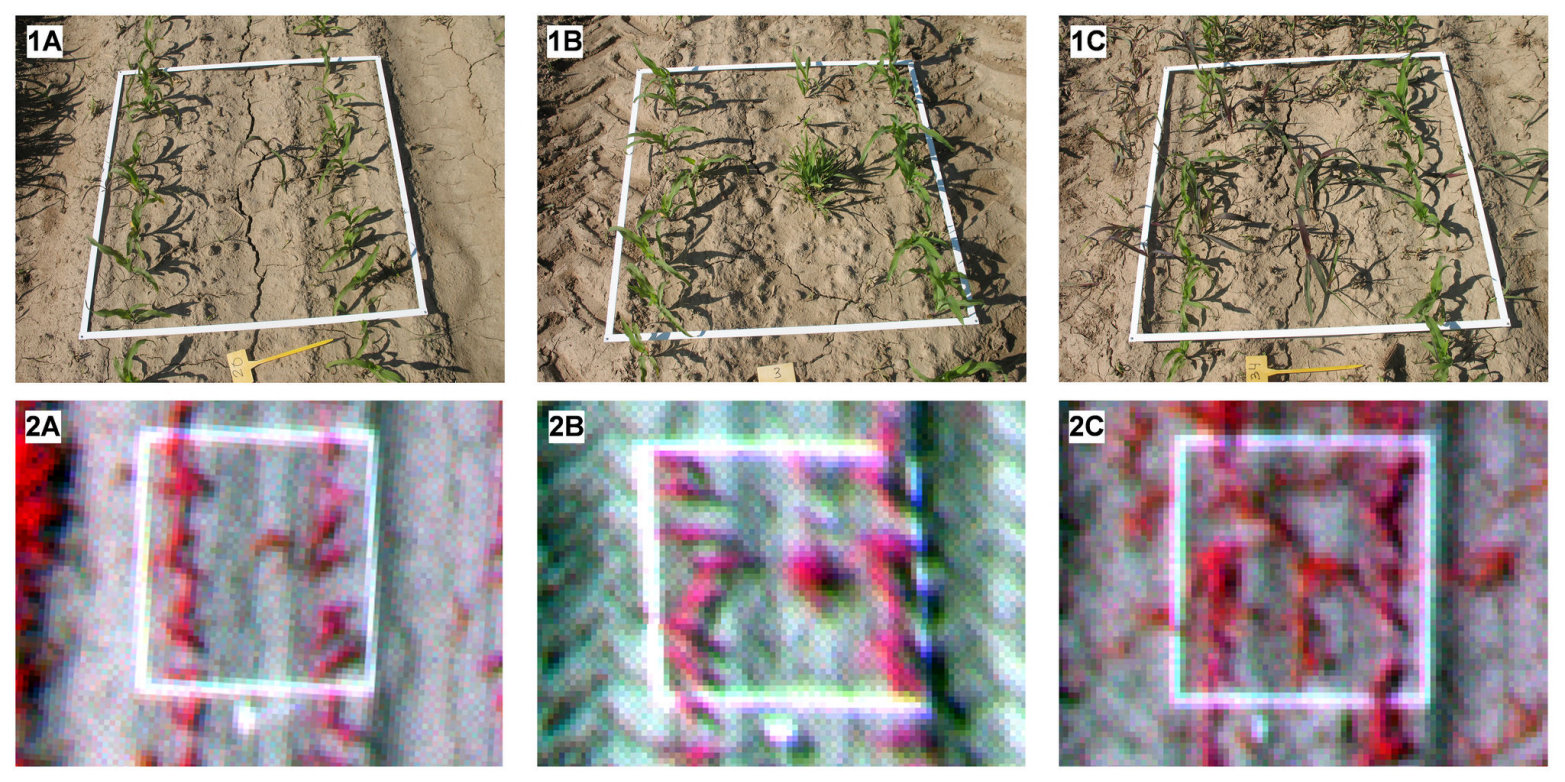
On-ground photographs (1) and UAV images (2) of the 1x1-m frames used in the ground-truth sampling of three different categories of weed coverage: *a*) low, *b*) moderate, and *c*) high.

The rule set algorithm was created and configured using two of the aerial images and was tested using the rest of the images. To evaluate the results of the algorithm, a systematic on-ground sampling procedure was conducted during the UAV flight. The sampling consisted of placing 28 square white frames, 1x1 m in size, throughout the studied surface (Figure 5). The distribution of the samples was representative of the distribution of weed coverage levels in the experimental field. Weed mapping is considered a more complicated task in cases of low and moderate levels of weed infestation (greater confusion is possible due to the presence of bare soil) than in cases of high levels of weed infestation (at which bare soil has a minor influence) or weed-free zones (with no influence of weeds). For this reason, the sampling frames were primarily located in zones with low and moderate weed coverage levels rather than in weed-free zones or in zones with high or very high infestation levels. 

Every frame was georeferenced with a GPS and photographed to compare on-ground weed infestation (observed weed coverage) with the outputs of the image classification process (estimated weed coverage). Weed coverage in the on-ground photographs was extracted through the application of a specific greenness index that accentuates the green color of the vegetation [27]. After a visual assessment of several indices, the excess green index [18,28] was selected for use and applied to the photographs. Next, pixels with values greater than zero were classified as vegetation (weed and crop), and finally, weed pixels were isolated by manually masking crop row areas. 

The fractions of weed area in the on-ground and aerial images were converted to percentages of the total area within every frame and were compared using a 1:1 line, which should have a correspondence of 1 in an ideal situation. This correspondence was evaluated by calculating the slope, the intercept and the coefficient of determination (R^2^) of a linear regression model. The root mean square error (RMSE) was also calculated as an additional measure of the overall error of the estimations. 

The accuracy of the classified images was also quantified by calculating the confusion matrix between weed mapping outputs and weed coverage in all the sampling frames grouped in the three categories (low, moderate and high weed densities) previously defined. The confusion matrix quantifies the overall accuracy (OA) of the classification, as well as its omission (OE) and commission (CE) errors in each category [29].

## Results and Discussion

### 1: Weed map information provided by the OBIA procedure

An advantage of the OBIA procedure, compared to traditional pixel-based methodologies, is its ability to compute multiple data and statistics derived from the image analysis and classification. Moreover, this information can be exported in several file formats, e.g., vector, image, ASCII, tables, etc. The algorithm developed in this study can compute and export information at several levels, depending on its position in the segmentation hierarchy, as described below. 

#### Whole field: upper segmentation level

Global information for the crop field, including field dimensions, number of crop rows, crop row orientation, average crop row separation, weed-free area and total area of each weed coverage category, was computed at the upper segmentation level. A vector shapefile with the limits of the field and a georeferenced image file of the gridded weed map were also produced, as well as other image files of intermediate classification outputs, if required. The global data computed for the experimental field are given in table 1. The experimental field occupied 1.4 ha and had 142 crop rows approximately 140 m in length, separated from each other by 0.70 m on average. The area free of weeds was 23%, and the area with low weed coverage (<5% of weeds) was 47%, indicating a high potential for reducing herbicide applications or other weed operations in this field.

**Table 1:** Global information on the whole experimental field computed according to the OBIA procedure at the upper segmentation level.

Global Feature	Value
*Field features*	
Area (m^2^)	14,000
Perimeter length (m)	480
Maximum length (m)	140
Minimum length (m)	100
Lat coordinate of the field center (°)	40.320 N
Lon coordinate of the field center (°)	3.477 W
*Crop row features*	
Number of rows (n)	142
Average row orientation (°)	32
Maximum row length (m)	140
Minimum row length (m)	140
Average distance between rows (m)	0.70
*Weed map features*	
Number of grid units (n)	19,880
Grid units free of weeds (n)	4,572
Grid units with weeds (n)	15,308
Area of grid units free of weeds (m^2^,%)	3,258 (23%)
Area of grid units with weeds (m^2^,%)	10,742 (77%)
Area with low weed coverage (<5%) (m^2^,%)	6,618 (47%)
Area with moderate weed coverage (5-20%) (m^2^,%)	3,230 (23%)
Area with high weed coverage (>20%) (m^2^,%)	894 ( 7%)

#### Crop row structure: Intermediate segmentation level

Detailed information on each inter-row unit, including the identification number (automatically assigned), the geographic coordinates of the row extremes, the length and width, the percentage of area free of weeds, and the percentage of each category of weed coverage considered, was produced at the intermediate segmentation level. An example of crop row data computed for the experimental field is given in table 2. Among the rows indicated, weeds were found in 100% of the grid units of row 141, which had 10% weed infestation. In contrast, row 1 only had 3% weed infestation and 57% of its grid units were free of weeds. 

**Table 2:** Inter-row information for the experimental field computed by the OBIA procedure at the intermediate segmentation level.

	Coordinates													
	Start			End			Size (m)			# Weed-infested grid units				
Row ID	Lat (40°N)	Lon (3°W)		Lat (40°N)	Lon (3°W)		Length	Width		Weed-free	Low (<5%)	Moderate (5–20%)	High (>20%)	Total
1	19´ 13.17”	28´ 38.93”		19´ 17.00”	28´ 35.72”		140	0.70		57	46	7	0	3
2	19´ 13.15”	28´ 38.90”		19´ 16.97”	28´ 35.69”		140	0.70		29	50	14	7	6
3	19´ 13.14”	28´ 38.86”		19´ 16.95”	28´ 35.65”		140	0.68		21	39	29	11	8
….	….	….		….	….		….	….		….	….	….	….	….
141	19´ 11.55”	28´ 35.29”		19´ 15.43”	28´ 32.03”		140	0.75		0	43	53	4	10
142	19´ 11.54”	28´ 35.26”		19´ 15.45”	28´ 32.06”		140	0.69		50	27	15	8	6

#### Weed infestation in grid units: lower segmentation level

Detailed information on each grid unit, including the identification number, geographic coordinates, dimensions, relative position within the crop row, distance to the start and the end of the crop row, weed coverage percentage and weed coverage category, was produced at the lower segmentation level. A list of the data computed in every grid unit of the experimental field is given in table 3. Among the grid units indicated, the highest weed coverage was measured in grid unit 3 (22%), located two meters from the beginning of row 1. In contrast, grid unit 1 was free of weeds.

**Table 3:** Grid information for the experimental field computed by the OBIA procedure at the lower segmentation level.

	Coordinates			Dimensions (m)			Position in row				Weed coverage	
Grid ID	Lat (40°N)	Lon (3°W)		Length	Width		Row ID	Distance to start (m)	Distance to end (m)		% of Weeds	Weed category
1	19´ 13.17”	28´ 38.93”		1	0.70		1	0	140		0	Weed-free
2	19´ 13.20”	28´ 38.90”		1	0.70		1	1	139		3	Low
3	19´ 13.23”	28´ 38.87”		1	0.70		1	2	138		22	High
….	….	….		….	….		….	….	….		….	….
19879	19´ 15.40”	28´ 32.05”		1	0.69		140	139	1		7	Moderate
19880	19´ 11.54”	28´ 35.26”		1	0.69		140	140	0		4	Low

The OBIA procedure generated a geo-referenced weed map that can be converted into a prescription herbicide application map and can then be transferred to machinery embedded with technologies for practical application of site-specific weed control strategies. The information provided in tables 1, 2 and 3 can be utilized by decision-making systems to calculate herbicide requirements or other weed operations in the field for the purposes of optimizing weeding machinery path planning and estimating the overall cost of weed management operations in advance [30]. Moreover, multi-temporal analysis of abundance and distribution of weeds within the same field is very helpful in studies of weed population dynamics and weed–crop interactions (e.g., crop yield losses).

### The evaluation of the weed map

**Figure 6:**
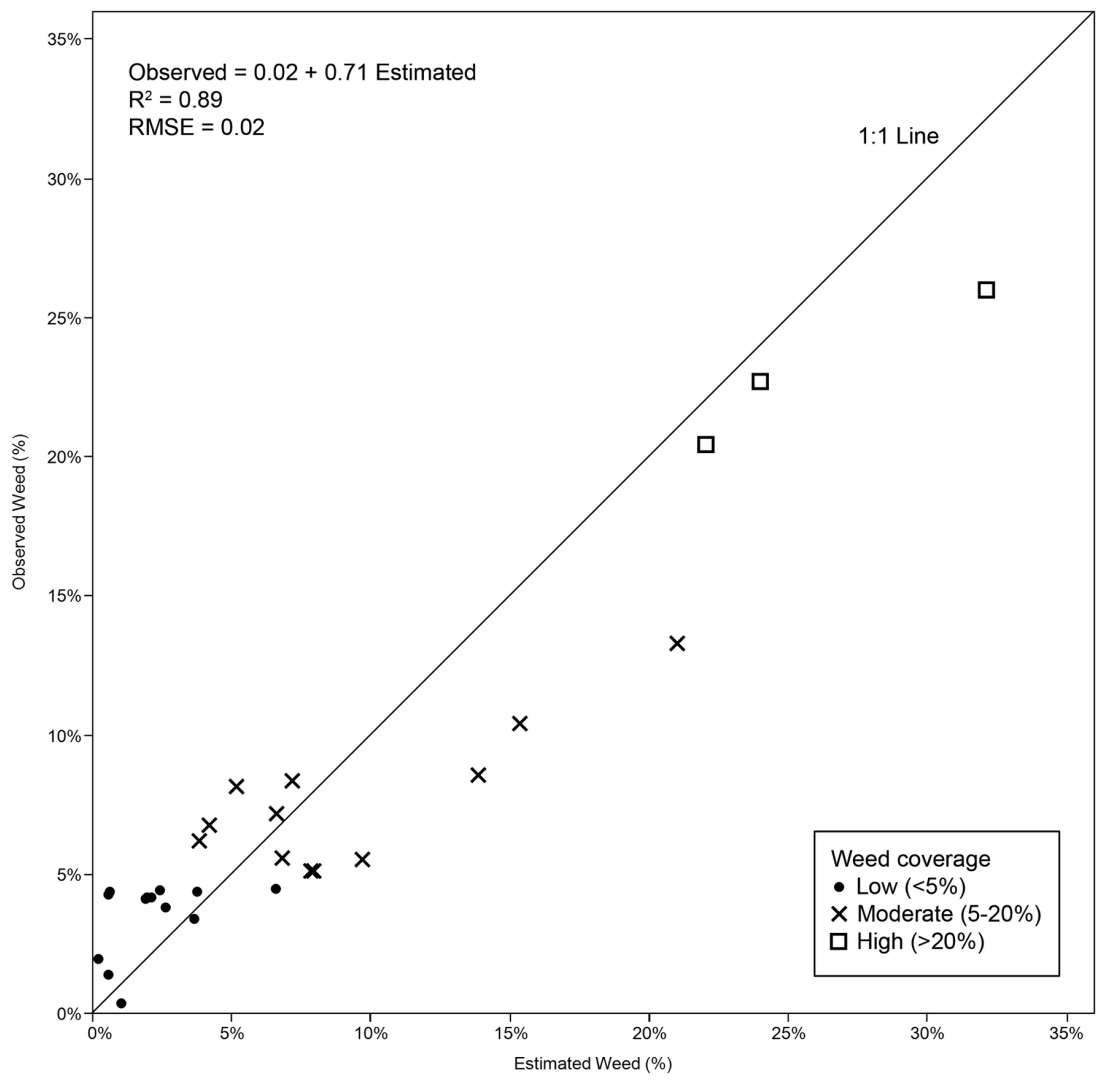
Observed and estimated weed coverage (%) inside the sampling frames from on-ground photographs and UAV image analysis, respectively.

The algorithm developed in this study identified and counted the rows in the training images with 100% accuracy and only had minor errors in classifying short rows located in the corners of some testing images. The definition of the longitudinal edge of the crop rows was strongly affected by the presence of weed plants very close to or within the crop rows. The accuracy of the methodology was evaluated by comparing the estimation of weed coverage derived from the UAV image classification and the values observed in the on-ground sampling photographs (Figure 6). The relationship between the estimated and observed weed densities was highly satisfactory, with a coefficient of determination of R^2^=0.89 and an RMSE=0.02, indicating good agreement in the three categories considered. 

At low weed coverage, most values were located above the 1:1 line, indicating some degree of overestimation of the weed infestation. From an agronomical perspective, this pattern of results is not adverse because it reduces the chance of missing isolated weeds. That is, it takes into account the fact that farmers might choose to treat weed-free zones, rather than assume the risk of allowing weeds to go untreated [31]. In contrast, the OBIA procedure slightly underestimated weed infestation at moderate and high weed densities, which is less important if it is corrected in the design of the herbicide prescription maps [32]. 

The weed map, with weed infestation levels classified in three categories, was also evaluated using the confusion matrix shown in table 4. The matrix indicates an overall accuracy of 86% and a kappa index of 0.76. The classification was over grid units, not over pixels, so the OA was the percentage of frames correctly classified (the number correct frames as a percentage of the total number of sampling frames). Confusion between frames was minor and only occurred between consecutive categories. The matrix also indicates the omission and commission errors in each category. OE indicates the proportion of frames with an observed weed coverage that was misclassified as being of a different coverage, and CE indicates the proportion of frames classified with levels of weed coverage that really correspond to other levels of coverage. As previously mentioned, only errors of underestimation of the weed category are important from the perspective of weed control [7], e.g., reporting 0% at low and high weed densities and reporting 17% of the frames at moderate weed coverage. 

**Table 4:** Classification matrix for three categories of weed coverage by comparing ground-truth weed sampling and the weed map derived from the UAV image classification.

Ground-truth weed sampling	UAV weed map					
	Low (<5%)	Moderate (5–20%)	High (>20%)	Number of frames	Omission Error	Underestimation Error
Low (<5%)	**12**	1		13	8%	0%
Moderate (5–20%)	2	**9**	1	12	25%	17%
High (>20%)			**3**	3	0%	0%
Number of frames	14	10	4	28		
Commission Error	15%	10%	25%			

Correct classifications are shown in bold.

Overall accuracy = 86%, Kappa index = 0.76

## CONCLUSIONS

An unmanned aerial vehicle and a six-band multispectral camera were used to collect remote images of a maize field in the early season for the purpose of generating weed maps for further early SSWM. A robust and automated OBIA procedure was developed for the automatic discrimination of crop rows and weeds in georeferenced and 2-cm spatial resolution remote images. The task was complex due to both the spectral properties and general appearance of weeds and crop plants are very similar in their early growth stages, and due to the difficulties created by variability and changing conditions in natural crop fields. The algorithm efficiently identified all the crop rows based on their linear pattern and on the contextual features of the vegetation objects that belong to the rows. Weed plants located in the inter-row area were then distinguished from crop plants on the basis of their relative positions with respect to the crop rows. Lastly, the weed cover percentages in three categories were determined to generate a weed map in a grid framework. The algorithm yielded very satisfactory results in most cases. 

The OBIA procedure computes multiple data and statistics derived from the image analysis and the classification outputs that can be exported in image, vector and table file formats. The tables and weed map provided helpful information that can be used in decision-making systems to calculate herbicide requirements and estimate the overall cost of weed management operations.

The combination of ultra-high-spatial-resolution UAV remote images and the OBIA procedure developed in this study permits the generation of weed maps in early maize crops for use in planning the application of in-season weed control measures, which has not been possible previously with traditional airborne or satellite images. This technology can help in the implementation of the European legislation for the sustainable use of pesticides, which promotes reductions in herbicide applications and the utilization of doses appropriate to the levels of weed infestation present.
